# Supramolecular Strategy Effects on Chitosan Bead Stability in Acidic Media: A Comparative Study

**DOI:** 10.3390/gels5010011

**Published:** 2019-02-25

**Authors:** Andrew J. Worthen, Kelly S. Irving, Yakov Lapitsky

**Affiliations:** Department of Chemical Engineering, University of Toledo, Toledo, OH 43606, USA; andrew.worthen@utoledo.edu (A.J.W.); kelly.irving@utoledo.edu (K.S.I.)

**Keywords:** chitosan, gel beads, stability, dissolution

## Abstract

Chitosan beads attract interest in diverse applications, including drug delivery, biocatalysis and water treatment. They can be formed through several supramolecular pathways, ranging from phase inversion in alkaline solutions, to the ionic crosslinking of chitosan with multivalent anions, to polyelectrolyte or surfactant/polyelectrolyte complexation. Many chitosan bead uses require control over their stability to dissolution. To help elucidate how this stability depends on the choice of supramolecular gelation chemistry, we present a comparative study of chitosan bead stability in acidic aqueous media using three common classes of supramolecular chitosan beads: (1) alkaline solution-derived beads, prepared through simple precipitation in NaOH solution; (2) ionically-crosslinked beads, prepared using tripolyphosphate (TPP); and (3) surfactant-crosslinked beads prepared via surfactant/polyelectrolyte complexation using sodium salts of dodecyl sulfate (SDS), caprate (NaC_10_) and laurate (NaC_12_). Highly variable bead stabilities with dissimilar sensitivities to pH were achieved using these methods. At low pH levels (e.g., pH 1.2), chitosan/SDS beads were the most stable, requiring roughly 2 days to dissolve. In weakly acidic media (at pH 3.0–5.0), however, chitosan/TPP beads exhibited the highest stability, remaining intact throughout the entire experiment. Beads prepared using only NaOH solution (i.e., without ionic crosslinking or surfactant complexation) were the least stable, except at pH 5.0, where the NaC_10_ and NaC_12_-derived beads dissolved slightly faster. Collectively, these findings provide further guidelines for tailoring supramolecular chitosan bead stability in acidic media.

## 1. Introduction

Chitosan is a bioderived polysaccharide that is produced through the deacetylation of chitin [1,2]. Due to its low toxicity [3,4] and ability to form an assortment of useful intermolecular complexes [5,6,7], chitosan attracts widespread interest in biomedical [8,9], biotechnological [10,11], environmental [12,13], food [14,15], and home and personal care [2] technologies. Chemically, chitosan is a random copolymer of β-(1-4)-linked d-glucosamine and *N*-acetyl-d-glucosamine monomer units, whose fraction of d-glucosamine units is known as its degree of deacetylation (DD) [1,2]. The ionizable d-glucosamine amine groups have an effective p*K_a_* of *ca.* 6.0–6.5, above which chitosan is typically insoluble, and below which chitosan is soluble [1,16].

When its amine groups are protonated, chitosan becomes a polyelectrolyte that can be assembled into diverse, gel-like soft materials [5,6,8,9]. These range from films and coatings [17,18], to fibers [19,20], colloidal particles [8,21,22], and macroscopic gels [9,23] and beads [13,24,25,26]. These materials can be prepared using diverse supramolecular strategies, which include ionic crosslinking of chitosan with multivalent ions [18,21,26], complexation of chitosan with anionic surfactants [13,19,24] or polymers [9,20,22,23], and precipitation of chitosan in alkaline solutions [11,13].

A key property that determines the performance of these chitosan-based materials is their degradation stability, which can be limited by either hydrolytic degradation of the chitosan chains [27,28,29] or simple dissolution [18,30,31,32,33]. Under dissolution-free conditions, which typically arise at neutral or alkaline pH (where chitosan is insoluble), or situations where the chitosan is essentially irreversibly crosslinked, the degradation of chitosan-based materials is controlled by hydrolytic chain scission [27,34]. The rate of this scission varies with the chitosan DD and, because it is slow for highly deacetylated chitosan, chitosan-based materials can (at least at near-neutral pH) remain intact over many weeks or months [27,28,29,35]. Conversely, when chitosan-based materials are prepared through reversible supramolecular crosslinking/association (using some of the strategies described above) and stored under acidic conditions (under which chitosan is soluble), chitosan chain scission tends not to be the stability-limiting factor. Instead, the dissolution stability in these cases depends on the supramolecular association strength (either between the chitosan chains or the chitosan and crosslinker [7,18,30,36]) and, in cases where a physical crosslinker is used, the crosslinker content within the chitosan network [26,32]. Previous stability studies revealed the association strength and, consequently, the stability of supramolecular chitosan-based materials to depend on the chitosan DD [30,37], pH [18,26,32] and ionic strength [18,30] of the dissolution media, and the molecular structure of the crosslinking/gelling agent [7,18,38]. 

To date, there have been many studies examining the stability of ionically crosslinked nanoparticles [30,39], films [7,32], and beads [26,38], as well as some investigating gels formed through complexation of chitosan and its derivatives with oppositely charged polyelectrolytes [31,40] and surfactants [33,36]. Yet, aside from some reports comparing chitosan complexation with small multivalent ions with chitosan-based polyelectrolyte complex formation [31,40], and studying the effects of various covalent and ionic crosslink types [7,38], there is a dearth of studies that directly compare the stability of self-assembled chitosan gels prepared through disparate mechanisms (e.g., alkaline precipitation versus ionic crosslinking, and polyelectrolyte and surfactant/polyelectrolyte complexation). To partially address this and provide further guidelines for designing supramolecular chitosan complexes, here we investigate the stability of chitosan-based beads in acidic aqueous solutions using beads prepared by three different supramolecular mechanisms: (1) precipitation in alkaline solution, (2) complexation with anionic surfactant, and (3) ionic crosslinking with tripolyphosphate (TPP), which is a common (and highly potent) ionic chitosan crosslinker [7,8,26,38]. Specifically, we focus on acidic conditions (pH = 1.2–5.0), which exist in many common chitosan applications (e.g., pharmaceutics, food and water treatment [7,12,15]) and at which chitosan is water-soluble. Using visual observations and stereomicroscopy to monitor bead dissolution, dissolution stabilities of the above bead types (i.e., their dissolution times and changes in external appearance) are systematically analyzed and compared.

## 2. Results and Discussion

### 2.1. Alkaline Solution-Derived Beads

Supramolecular chitosan beads were generated via dropwise addition of a 3 wt% aqueous chitosan solution (pH. 5.0) into either 0.1 M NaOH or 1 wt% TPP, sodium caprate (NaC_10_), sodium laurate (NaC_12_) or sodium dodecyl sulfate (SDS) solutions through a 20-gauge syringe needle and equilibrating the resulting mixtures (which successfully formed millimeter-scale spherical beads) at 30 °C for at least 24 h. Here, the 1 wt% ionic crosslinker/surfactant concentrations were selected to remain fixed (so that their molecular structure alone was varied) and to safely not exceed the solubility limit of the least soluble surfactant, NaC_12_ [41]. Upon equilibration, single beads were placed into the dissolution media (10 mM of aqueous NaCl solutions at varying, 1.2–5.0 pH levels) and monitored for dissolution by: (1) imaging the beads via stereomicroscopy; and (2) visually determining the times at which their full dissolution occurred.

**Figure 1:**
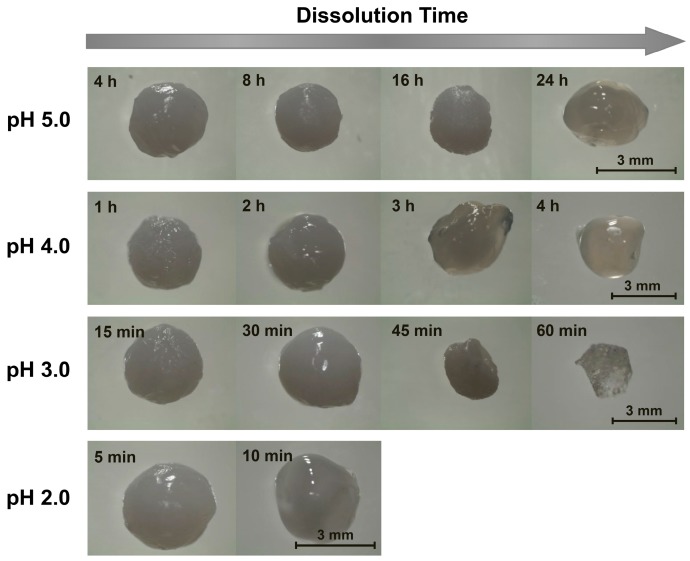
Representative images of alkaline solution-derived chitosan beads dissolving in pH 1.2–5.0 aqueous solutions ([NaCl] = 10 mM).

**Figure 2:**
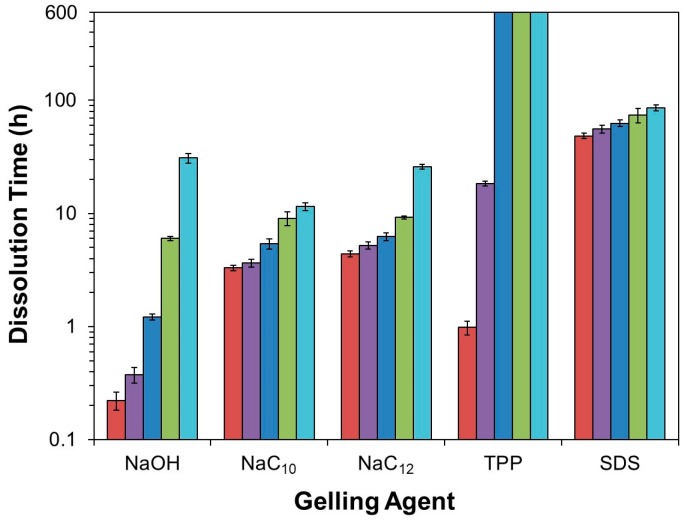
Summary of bead dissolution times in 10 mM aqueous NaCl solutions at pH (■) 1.2, (■) 2.0, (■) 3.0, (■) 4.0, and (■) 5.0, increasing in this plot from left to right. The bars reaching the top of the plot for the chitosan/TPP beads at pH 3.0–5.0 indicate that these beads remained intact throughout the entire experiment. For all other conditions, error bars representing one standard deviation in bead dissolution time are given (*n* = 6).

Since chitosan is soluble at acidic pH levels [1,16], beads formed through precipitation in NaOH solutions all eventually dissolved in the acidic dissolution media. The timescales of this dissolution, however, were highly pH-dependent. At pH 5.0 (the highest examined dissolution pH), the beads remained outwardly unchanged over the first several hours. After 8 h, however, the beads began to slowly shrink, and after 16 h, they began to deform (Figure 1). This deformation increased substantially after 24 h, whereupon the initially rigid (and highly opaque) beads became both soft and more translucent (Figure 1). Within a few hours of losing their rigidity, the beads started to rapidly shrink (due to their accelerated dissolution), until completely dissolving after roughly 30 h (Figure 2).

When the experiments were repeated in more acidic, pH 4.0 dissolution media, the dissolution time diminished sharply to approximately 6 h (Figure 2). Here, the beads began to deform and swell after just 3 h of dissolution, and started to become transparent and shrink after 4 h (Figure 1). When the dissolution media pH was decreased to pH 3.0, bead dissolution became even faster (occurring in just slightly over 1 h; Figure 2). The evolution in the bead morphology, however, was different than that in less acidic media. After appearing to remain unchanged over the first 15 min, the beads swelled slightly after 30 min, shrank and became less spherical after 45 min, and became almost flat and flake-like after 60 min (Figure 1).

In the limit of very low pH values of 2.0 and 1.2, the dissolution was accelerated to the point where it became difficult to image the dissolving beads. At pH 2.0, the beads began shrinking after 5 min and started to become more transparent after 10 min (Figure 1). After 15 min the beads became completely transparent, whereupon they were fully dissolved in slightly over 20 min (Figure 2). This rapid dissolution became even faster at pH 1.2, which was similar to the gastric environment encountered by orally administered capsules. The beads shrank substantially after only 1 min of dissolution, were almost completed dissolved after a few min, and were fully dissolved after about 10 min in the dissolution media (Figure 2). This ready dissolution of uncrosslinked chitosan beads was consistent with reports by Lipatova and Makharova, who showed chitosan flakes to dissolve in acetic acid solution over the timescale of hours [42], and by Wan Ngah et al., who reported uncrosslinked chitosan beads to dissolve in 5 vol% acetic acid solutions [43]. These rapid dissolution times show that alkaline solution-derived chitosan beads are highly unstable in the limit of low pH levels, such as those encountered by orally administered drug formulations in the gastric fluid, but can withstand mildly acidic media (such as may exist in food formulations or during bioprocessing) on the timescale of hours.

### 2.2. Tripolyphosphate-Crosslinked Beads

**Figure 3:**
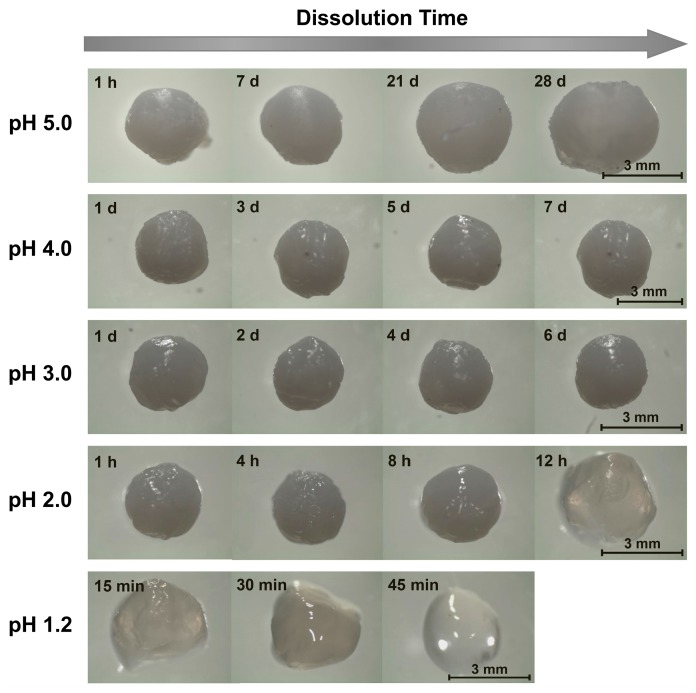
Representative images of chitosan/TPP beads dissolving in pH 1.2–5.0 aqueous solutions ([NaCl] = 10 mM).

Unsurprisingly, when the beads were ionically crosslinked with TPP, their stability to dissolution in acidic environments increased. In milder acidic media (3.0 ≤ pH ≤ 5.0), the beads persisted throughout the entire 7-day quantitative dissolution experiment (Figure 2) and exhibited almost no change in appearance (Figure 3). Yet, though this lack of change over 7 days indicates the beads to be much more stable in mildly acidic media than their alkaline solution-derived counterparts, it does not mean that the beads remain stable indefinitely. Indeed, imaging the beads placed in pH 5.0 solution over longer (28-day) timescales revealed that the beads began to swell (see Figure 3), which hinted at their slow dissociation and dissolution [31]. This progression likely reflects the slow leaching of crosslinking TPP ions [26,30] and indicates limits to the stability of these chitosan/TPP networks.

The bead stability was even more limited when the beads were placed in highly acidic pH 1.2–2.0 media. At pH 2.0, for instance, the opaque beads became both swollen and translucent after 12 h, and fully dissolved after roughly 18 h (Figure 2 and Figure 3). Similarly, at pH 1.2, the beads became transparent after just 45 min and completely dissolved in roughly 1 h. This sharp pH effect on the bead stability reflects the impact of pH on chitosan/TPP binding strength. TPP is a polyprotic acid (p*K_a,3_* = 2.8, p*K_a,4_* = 6.5, and p*K_a,5_* = 9.2 [44]) and, at low pH levels, becomes partially protonated. This protonation reduces TPP ionization and, accordingly, weakens its binding to cationic chitosan and causes the beads to dissolve in highly acidic media. This observation is qualitatively consistent with those reported by Jóźwiak et al., who reported chitosan/TPP beads to rapidly dissolve at pH 2.0 and (when the beads had a low TPP content) 3.0 [38], and by Mi et al., who reported the phosphorus elution to increase sharply at low (1.2–3.0) pH levels [26]. Yet, Mi et al. indicated chitosan/TPP beads formed at very high TPP contents (achieved using much more concentrated, 10 wt% TPP solutions at low pH levels) could be maintained intact for up to 1–2 days, even at pH 1.0 [26,40]. This was evidently because the initial TPP content within these beads was high enough (at least in a batch experiment without solvent replacement) to prevent bead swelling and dissolution, even when >30% of the TPP leached from the beads [26]. Thus, it may be possible to increase the bead dissolution times in Figure 2 by increasing their TPP content [26,32,40] or (since chitosan/TPP binding strength increases with the chitosan DD [30]) using chitosan with even higher DD values. Despite these possibilities, at the 1 wt% TPP concentrations (with no pH adjustments) used in the present work, ionic crosslinking with TPP stabilizes chitosan beads in moderately acidic media (e.g., at 3.0 ≤ pH ≤ 5.0), but provides only modest improvements in bead stability at the highly acidic pH of gastric fluid. 

### 2.3. Surfactant-Complexed Beads

Chitosan/surfactant bead stability was less sensitive to pH than that of alkaline solution-derived and TPP-crosslinked beads (see Figure 2) and, not surprisingly, this stability increased with the surfactant strength (i.e., its tendency to self-associate: SDS > NaC_12_ > NaC_10_ [45,46]). As the dissolution medium pH, for instance, was lowered from 5.0 to 1.2, the average dissolution times of chitosan/NaC_10_ beads only decreased from ~11–12 h to slightly over 3 h, while the stability of chitosan/NaC_12_ and chitosan/SDS beads decreased from roughly 1 day to about 4 h and from roughly 3.5 to 2 days, respectively. Throughout, the beads formed with stronger surfactants persisted longer, and the 1.8- to 5.9-fold reductions in bead stability as the dissolution medium pH was decreased from 5.0 to 1.2 were mild compared to the multiple-order-of-magnitude reduction exhibited by the alkaline solution-derived beads and chitosan/TPP beads (Figure 2). 

Beads formed from surfactants with longer, dodecyl aliphatic tails (i.e., NaC_12_ and SDS) formed more stable complexes with chitosan than the shorter-tailed NaC_10_. This improved stability was qualitatively consistent with previous work on the stability of quaternized chitosan/fatty acid complexes [36], where the increased stability of complexes formed with longer-tailed fatty acids was attributed to the favorable hydrophobic free energy of transfer of aliphatic CH_2_ groups into the hydrophobic environment of the surfactant/polyelectrolyte complex [36,47]. Since the same hydrophobic effect drives micelle formation, surfactant/polyelectrolyte binding strength tends to scale inversely with the surfactant critical micelle concentration (CMC) [47,48], and thus, surfactant/polyelectrolyte complexes prepared from surfactants with lower CMC values are generally more stable to dissolution. Accordingly, the qualitative trend in chitosan/surfactant bead stability shown in Figure 2 is consistent with that of the CMCs, where the CMC of SDS in deionized water (ca. 8 mM) is roughly three times lower than that of NaC_12_ (23–24 mM), and more than ten times lower than that of NaC_10_ (94–96 mM) [45,46]. Unlike the previous dissolution study (which was conducted in near-neutral phosphate-buffered saline [36]), however, the present study was performed under acidic conditions, where considerable protonation of fatty acid was expected [49]. This protonation likely further weakened fatty acid binding to the chitosan compared to the relatively pH-insensitive SDS. 

Interestingly, though surfactant/polyelectrolyte complexation stabilized the chitosan beads under most conditions, beads formed using weakly binding NaC_10_ and NaC_12_ dissolved slightly faster at pH 5.0 than beads prepared in NaOH solution (see Figure 2). This accelerated dissolution likely stemmed from: (1) a lower initial bead pH (as the 1 wt% fatty acid solution was less alkaline than the 0.1 M NaOH solution); and (2) a higher initial chitosan protonation state caused by the ionic chitosan/fatty acid complexation (which, along with the lower initial pH, reduced the amount of acid diffusion needed to acidify the beads and solubilize the chitosan). These pH and protonation effects evidently outweighed the added stabilization achieved through the weak chitosan/fatty acid complexation. 

**Figure 4:**
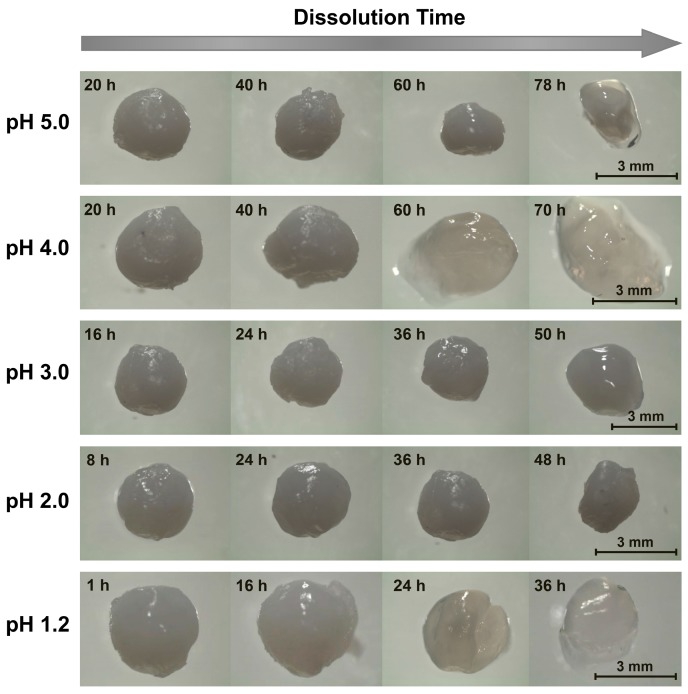
Representative images of chitosan/sodium salts of dodecyl sulfate (SDS) beads dissolving in pH 1.2–5.0 aqueous solutions ([NaCl] = 10 mM).

Another difference in the dissolution of chitosan/surfactant beads versus the other examined bead types was in the evolution in the bead appearance; specifically, in the outer shells of the surfactant/polyelectrolyte beads frequently delaminating from their internal layers. When chitosan/SDS beads were placed in pH 5.0 dissolution media, for instance, the beads remained relatively unchanged over 20 h of dissolution, but began to shrink after 40 h (see Figure 4). After 60 h, the beads shrank even more noticeably, and after 78 h, the outer shells of the beads became transparent and started delaminating (with the bead fully dissolving after 80–90 h). As the dissolution medium pH was reduced to 4.0, the delamination started to occur earlier, with some deformation occurring after 40 h, pealing of the outer shell noticed after 60 h, and full bead dissolution happening after 3 days (shortly before which the bead became completely transparent; data not shown). The delamination in each case likely reflected the layered structure of the surfactant/polyelectrolyte bead surfaces, which was revealed in earlier studies on their internal morphologies, where similar delamination, buckling, and/or loss of the outer shell during swelling and dissolution also occurred [50,51].

**Figure 5:**
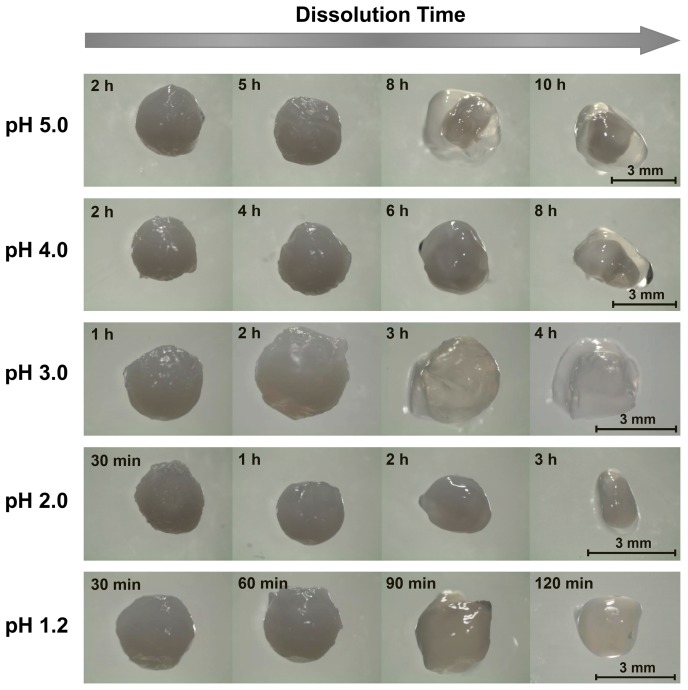
Representative images of chitosan/NaC_10_ beads dissolving in pH 1.2–5.0 aqueous solutions ([NaCl] = 10 mM).

Interestingly, at lower pH levels, the outer layer delamination was less evident (Figure 4). Despite remaining virtually unchanged in its first 16 h at pH 3.0, the beads began deforming after 24 h, and significantly softened after 36 h. This softening was followed by swelling and a further loss in rigidity after 50 h, and then dissolution after 63 h. As the dissolution pH was lowered further yet (to pH 2.0), the beads appeared to maintain their initial properties during the first 8 h, but began to swell after 24 h. After 36 h, they started to shrink (Figure 4), until ultimately dissolving after 50–60 h. Similar (albeit faster) dissolution trends occurred when the SDS was replaced with fatty acids. When NaC_10_-based beads, for instance, were placed in pH 5.0 media, the beads remained relatively unchanged for the first several hours of dissolution (Figure 5). When the beads were imaged again after 8 h of dissolution, however, a transparent outer layer formed. After 10 h, though it is not evident in Figure 5, the clear layer started separating from the rest of the bead; and after roughly 11–12 h, the beads completely dissolved. Similarly, at lower pH levels, this dissociation occurred more rapidly (with complete dissolution happening within just ~3 h at pH 1.2). Instead of opacity reduction and delamination initially occurring at the outside of the bead, however, bulk swelling and loss of opacity were more prevalent at lower pH levels, with surface layer delamination becoming less evident. Fairly similar morphological transitions were also seen for NaC_12_-based beads (data not shown), whose binding to chitosan was intermediate to that of the strongly binding SDS and weakly binding NaC_10_.

### 2.4. Further Discussion

Overall, this bead dissolution study demonstrates that: (1) though each of the examined supramolecular chitosan beads ultimately dissolve (or at least shows signs of dissociation) in acidic media, some of these beads are more stable than others; and (2) the order of this stability may depend on whether the media is weakly or strongly acidic. In weakly acidic media (e.g., at pH 5.0, which is not atypical for bioprocesses and food formulations), TPP-crosslinked beads are the most stable, remaining intact (albeit in a slightly more swollen state) for several weeks. In highly acidic environments (such as pH 1.2, which exists in gastric fluids), however, TPP becomes protonated and, due to its lower anionic charge and weaker binding, provides only a minor stabilizing effect under the conditions examined in this work. Further, though earlier publications by Mi et al. reported chitosan/TPP beads prepared using 10 wt% TPP at a low pH (which likely resulted in beads with a higher TPP content relative to those prepared in the present study) to remain stable even at pH 1.0 [26,40], these observations were made in experiments where, despite the beads remaining intact, considerable (>30%) TPP elution from the beads occurred [26]. These findings suggest that upon eluting this amount of TPP, an equilibrium TPP partitioning between the beads and batch of dissolution media was achieved; and, if a higher media-to-bead ratio was used (or if the dissolution media was regularly replaced as done in our work), further TPP elution and bead dissolution would have eventually occurred. Thus, though the beads prepared via the method of Mi et al. would likely have been more stable than those used here, the magnitude of this stabilization effect remains uncertain.

Unlike the chitosan/TPP gels, beads prepared through chitosan complexation with the strong anionic surfactant SDS are relatively insensitive to pH and require 2 days to fully dissolve at the gastric pH of 1.2. Thus, in the limit of low pH, chitosan/SDS beads are the most stable of the beads in our study (though, based on prior work by Mi et al. [31,40] and others [52,53], we expect that similar or greater stability at low pH might also be achieved using chitosan-based polyelectrolyte complexes, or possibly by increasing the chitosan-bound TPP content within the chitosan/TPP beads [26,40]). 

Weak anionic surfactants (NaC_10_ and NaC_12_) also generate beads whose dissolution stability is less sensitive to pH than that of beads formed in NaOH and TPP solutions. Due to their weaker binding to chitosan, however, they do not provide as strong of a stabilizing effect as SDS. Indeed, as discussed in Section 2.3, their use as the gelling agent can reduce bead stability in weakly acidic media (as seen at pH 5.0), and could perhaps be used as a strategy to accelerate (rather than decelerate) bead dissolution under these conditions. Conversely, uncrosslinked chitosan beads (obtained by gelling chitosan in alkaline solution) remain stable for hours in mildly acidic solutions, but dissolve within minutes in highly acidic conditions. Since acid and enzymatically catalyzed hydrolysis of highly deacetylated chitosan (such as used in this work) is expected to be slow [27,28,29], bead stabilities over timescales ranging from minutes to multiple days (or even weeks/months) can likely be achieved through a judicious selection of supramolecular gelation pathways. 

## 3. Conclusions

Stability of chitosan beads formed through alkaline precipitation, ionic crosslinking, and surfactant/polyelectrolyte complexation in acidic dissolution media was compared. The stability of each of these bead types decreased with the increasing acidity of the dissolution media. Under the conditions examined, the uncrosslinked beads remain stable for time periods ranging from minutes to hours, depending on the acid concentration. Chitosan/TPP beads (when prepared using TPP in a modest charge excess at its natural pH) are highly sensitive to pH, dissolving within an hour at low pH levels (e.g., at the gastric fluid pH of 1.2), but persisting over at least weeks at the mildly acidic pH of 5.0. Conversely, anionic surfactant-associated chitosan beads are the least sensitive to pH, remain stable in acidic media for up to days at all of the examined pH levels, and have dissolution properties that can be extensively tuned by varying the surfactant/polyelectrolyte binding strength (by, for instance, changing the anionic surfactant used). Thus, the type of bead that provides the highest stability may depend on whether the dissolution media is weakly acidic (such as may be encountered in food formulations or bioprocessing, where chitosan/TPP beads were the most stable) or strongly acidic (as in the case of gastric fluid where, out of the beads used in this work, chitosan/SDS beads were the most stable). Overall, these findings extend existing guidelines on tuning the stability of supramolecular chitosan gel beads to their diverse technological applications.

## 4. Materials and Methods

### 4.1. Materials

All experiments were performed using Millipore Direct-Q 3 deionized water (18.2 MΩ·m resistivity). Chitosan (90% DD, as determined by pH titration and viscosity-average molecular weight = 120 kDa [36]), TPP (sodium salt) and NaC_12_ were purchased from Sigma-Aldrich (St. Louis, MO, USA). SDS was obtained from MP Biomedicals (Solon, OH, USA), HCl was bought from both Sigma-Aldrich (St. Louis, MO, USA) and VWR International, LLC (West Chester, PA, USA), and NaC_10_ was purchased from Spectrum (New Brunswick, NJ, USA). NaCl and NaOH were obtained from Fisher Scientific (Fair Lawn, NJ, USA). All materials were used as received.

### 4.2. Bead Preparation

Chitosan solution (3 wt%) at pH 5.0 was prepared by dissolving chitosan in water, while adding HCl until the pH was 5.00 ± 0.05. The solution was then equilibrated for 12–16 h at 30 °C, whereupon the pH was checked one final time to ensure that it remained stable. The chitosan solution was then loaded into 3 mL BD Luer-Lok tip syringes, discarding approximately 10% of the solution at the bottom of the bottle, to avoid loading any undissolved impurities. 

To form the beads, the 3 wt% chitosan solution was added dropwise (at a rate of roughly 30 drops/min) through a 20-gauge syringe needle into gently agitated solutions of either 0.1 M of NaOH, 1 wt% TPP, 1 wt% NaC_10_, 1 wt% NaC_12_ or 1 wt% SDS. All solutions were stored at 30 °C prior to bead preparation to maintain their temperature above the Krafft point of the least soluble surfactant (NaC_12_ [41]) and ensure that all of the gelling agents remained soluble prior to the chitosan addition. The volume ratio between the receiving (NaOH, TPP, NaC_10_, NaC_12_ or SDS) solutions and the chitosan solution was maintained above 10:1 throughout the dropwise addition process, which in the case of ionically associating TPP, NaC_10_, NaC_12_ and SDS, kept the gelling agent:chitosan charge ratios above 1.7:1, 3.1:1, 2.7:1 and 2.1:1, respectively (i.e., the anionic gelling agent was always in excess). Once the beads were formed, the resulting mixtures were stored in a 30 °C water bath for at least 24 h (until the beads were separated from their surrounding solvent for further analysis).

### 4.3. Dissolution Experiments

To test their dissolution stability, chitosan beads (one per each trial) were equilibrated at room temperature in an excess of 10 mM NaCl solution pH adjusted to 1.2, 2.0, 3.0, 4.0 or 5.0 using HCl. Dissolution was considered complete when no visible material remained in the dissolution medium. To prepare the beads for dissolution, they were removed from the solutions in which they were formed and rinsed with 5–8 mL of water before being placed into 1 L of dissolution medium. Dissolution experiments were then performed by allowing one bead to dissolve in a 1 L Pyrex Vista beaker, agitated with a 5/16” octagonal stir bar at 500 rpm. Besides recording the times required for the beads to fully dissolve, evolutions in their size and morphology during dissolution were tracked by periodically imaging them with a Leica EZ4 D (Buffalo Grove, IL, USA) digital stereomicroscope. During each imaging step, the beads were removed from the dissolution medium using a wire mesh, and imaged after removing the surface water with a VWR (West Chester, PA, USA) light duty tissue wiper. The imaged beads were then returned to the dissolution media. For experiments lasting longer than 24 h, the dissolution medium was replaced daily. Each dissolution experiment was reproduced six times. 

## References

[1] Pillai C.K.S., Paul W., Sharma C.P. (2009). Chitosan and chitosan polymers: Chemistry, solubility and fiber formation. Prog. Polym. Sci..

[2] Rinaudo M. (2006). Chitin and chitosan: Properties and applications. Prog. Polym. Sci..

[3] Tapola N.S., Lyyra M.L., Kolehmainen R.M., Sarkkinen E.S., Schauss A.G. (2008). Safety aspects and cholesterol-lowering efficacy of chitosan tablets. J. Am. Coll. Nutr..

[4] VandeVord P.J., Matthew H.W., DeSilva S.P., Mayton L., Wu B., Wooley P.H. (2002). Evaluation of the biocompatibility of a chitosan scaffold in mice. J. Biomed. Mater. Res..

[5] Chiappisi L., Gradzielski M. (2015). Co-assembly in chitosan–surfactant mixtures: thermodynamics, structures, interfacial properties and applications. Adv. Coll. Int. Sci..

[6] Luo Y., Wang Q. (2014). Recent development of chitosan-based polyelectrolyte complexes with natural polysaccharides for drug delivery. Int. J. Biol. Macromol..

[7] Shu X., Zhu K. (2002). Controlled drug release properties of ionically cross-linked chitosan beads: The influence of anion structure. Int. J. Pharm..

[8] Garcia-Fuentes M., Alonso M.J. (2012). Chitosan-based drug nanocarriers: Where do we stand?. J. Controll. Release.

[9] Madihally S.V., Matthew H.W.T. (1999). Porous chitosan scaffolds for tissue engineering. Biomaterials.

[10] Krajewska B. (2004). Application of chitin-and chitosan-based materials for enzyme immobilizations: A review. Enzyme Microb. Technol..

[11] Wahba M.I. (2018). Sodium bicarbonate-gelled chitosan beads as mechanically stable carriers for the covalent immobilization of enzymes. Biotechnol. Prog..

[12] Wang J., Chen C. (2014). Chitosan-based biosorbents: Modification and application for biosorption of heavy metals and radionuclides. Bioresour. Technol..

[13] Chatterjee S., Lee D.S., Lee M.W., Woo S.H. (2009). Congo red adsorption from aqueous solutions by using chitosan hydrogel beads impregnated with nonionic or anionic surfactant. Bioresour. Technol..

[14] Aider M. (2010). Chitosan application for active bio-based films production and potential in the food industry. LWT-Food Sci. Technol..

[15] Coma V., Martial-Gros A., Garreau S., Copinet A., Salin F., Deschamps A. (2002). Edible antimicrobial films based on chitosan matrix. J. Food Sci..

[16] Sorlier P., Denuziere A., Viton C., Domard A. (2001). Relation between the degree of acetylation and the electrostatic properties of chitin and chitosan. Biomacromolecules.

[17] Meng S., Liu Z., Shen L., Guo Z., Chou L.L., Zhong W., Du Q., Ge J. (2009). The effect of a layer-by-layer chitosan–heparin coating on the endothelialization and coagulation properties of a coronary stent system. Biomaterials.

[18] Shu X.Z., Zhu K.J. (2002). The influence of multivalent phosphate structure on the properties of ionically cross-linked chitosan films for controlled drug release. Eur. J. Pharm. Biopharm..

[19] Mendes A.C., Gorzelanny C., Halter N., Schneider S.W., Chronakis I.S. (2016). Hybrid electrospun chitosan-phospholipids nanofibers for transdermal drug delivery. Int. J. Pharm..

[20] Toivonen M.S., Kurki-Suonio S., Wagermaier W., Hynninen V., Hietala S., Ikkala O. (2017). Interfacial polyelectrolyte complex spinning of cellulose nanofibrils for advanced bicomponent fibers. Biomacromolecules.

[21] Calvo P., Remunan-Lopez C., Vila-Jato J.L., Alonso M.J. (1997). Novel hydrophilic chitosan-polyethylene oxide nanoparticles as protein carriers. J. Appl. Polym. Sci..

[22] Saether H.V., Holme H.K., Maurstald G., Smidsrod O., Stokke B.T. (2008). Polyelectrolyte complex formation using alginate and chitosan. Carbohydr. Polym..

[23] Shchipunov Y., Sarin S., Kim I., Ha C.-S. (2010). Hydrogels formed through regulated self-organization of gradually charging chitosan in solution of xanthan. Green Chem..

[24] Babak V.G., Merkovich E.A., Desbrieres J., Rinaudo M. (2000). Formation of an ordered nanostructure in surfactantpolyelectrolyte complexes formed by interfacial diffusion. Polym. Bull..

[25] Yu L., Liu X., Yuan W., Brown L.J., Wang D. (2015). Confined flocculation of ionic pollutants by poly (L-dopa)-based polyelectrolyte complexes in hydrogel beads for three-dimensional, quantitative, efficient water decontamination. Langmuir.

[26] Mi F.L., Shyu S.S., Lee S.T., Wong T.B. (1999). Kinetic study of chitosan-tripolyphosphate complex reaction and acid-resistive properties of the chitosan-tripolyphosphate gel beads prepared by in-liquid curing method. J. Polym. Sci. B Polym. Phys..

[27] Freier T., Koh H.S., Kazazian K., Shoichet M.S. (2005). Controlling cell adhesion and degradation of chitosan films by N-acetylation. Biomaterials.

[28] Tomihata K., Ikada Y. (1997). In vitro and in vivo degradation of films of chitin and its deacetylated derivatives. Biomaterials.

[29] Vårum K., Ottøy M., Smidsrød O. (2001). Acid hydrolysis of chitosans. Carbohydr. Polym..

[30] Huang Y., Cai Y., Lapitsky Y. (2015). Factors affecting the stability of chitosan/tripolyphosphate micro- and nanogels: Resolving the opposing findings. J. Mater. Chem. B.

[31] Mi F.L., Shyu S.S., Kuan C.Y., Lee S.T., Lu K.T., Jang S.F. (1999). Chitosan–polyelectrolyte complexation for the preparation of gel beads and controlled release of anticancer drug. I. Effect of phosphorous polyelectrolyte complex and enzymatic hydrolysis of polymer. J. Appl. Polym. Sci..

[32] Remunan-Lopez C., Bodmeier R. (1997). Mechanical, water uptake and permeability properties of crosslinked chitosan glutamate and alginate films. J. Controll. Release.

[33] Worthen A.J., Lapitsky Y. (2011). Stabilization of bioderived surfactant/polyelectrolyte complexes through surfactant conjugation to the biopolymer. Colloid Polym. Sci..

[34] Sashiwa H., Saimoto H., Shigemasa Y., Ogawa R., Tokura S. (1990). Lysozyme susceptibility of partially deacetylated chitin. Int. J. Biol. Macromol..

[35] Kim H., Tator C.H., Shoichet M.S. (2011). Chitosan implants in the rat spinal cord: biocompatibility and biodegradation. J. Biomed. Mater. Res. A.

[36] Lapitsky Y., Zahir T., Shoichet M.S. (2008). Modular biodegradable biomaterials from surfactant and polyelectrolyte mixtures. Biomacromolecules.

[37] Rusu-Balaita L., Desbrieres J., Rinaudo M. (2003). Formation of a biocompatible polyelectrolyte complex: Chitosan-hyaluronan complex stability. Polym. Bull..

[38] Jóźwiak T., Filipkowska U., Szymczyk P., Rodziewicz J., Mielcarek A. (2017). Effect of ionic and covalent crosslinking agents on properties of chitosan beads and sorption effectiveness of Reactive Black 5 dye. React. Funct. Polym..

[39] Morris G.A., Castile J., Smith A., Adams G.G., Harding S.E. (2011). The effect of prolonged storage at different temperatures on the particle size distribution of tripolyphosphate (TPP)—chitosan nanoparticles. Carbohydr. Polym..

[40] Mi F.L., Shyu S.S., Wong T.B., Jang S.F., Lee S.T., Lu K.T. (1999). Chitosan–polyelectrolyte complexation for the preparation of gel beads and controlled release of anticancer drug. II. Effect of pH-dependent ionic crosslinking or interpolymer complex using tripolyphosphate or polyphosphate as reagent. J. Appl. Polym. Sci..

[41] McBain J., Sierichs W. (1948). The solubility of sodium and potassium soaps and the phase diagrams of aqueous potassium soaps. J. Am. Oil Chem. Soc..

[42] Lipatova I., Makarova L. (2008). Effect of hydroacoustic treatment on chitosan dissolution in aqueous acetic acid solutions. Russ. J. Appl. Chem..

[43] Wan Ngah W., Endud C., Mayanar R. (2002). Removal of copper (II) ions from aqueous solution onto chitosan and cross-linked chitosan beads. React. Funct. Polym..

[44] Beukenkamp J., Rieman W., Lindenbaum S. (1954). Behavior of the condensed phosphates in anion-exchange chromatography. Anal. Chem..

[45] Campbell A., Lakshminarayanan G. (1965). Conductances and surface tensions of aqueous solutions of sodium decanoate, sodium laurate, and sodium myristate, at 25 and 35. Can. J. Chem..

[46] Sjostrom J., Piculell L. (2001). Interactions between cationically modified hydroxyethyl cellulose and oppositely charged surfactants studied by gel swelling experiments—Effects of surfactant type, hydrophobic modification and added salt. Colloids Surf. A.

[47] Goddard E.D. (1986). Polymer surfactant interaction. 2. Polymer and surfactant of opposite charge. Colloids Surf..

[48] Malovikova A., Hayakawa K., Kwak J.C.T. (1984). Surfactant Polyelectrolyte interactions. 4. Surfactant chain-length dependence on the binding of alkylpyidinium cations to dextran sulfate. J. Phys. Chem..

[49] Kanicky J.R., Shah D.O. (2003). Effect of premicellar aggregation on the pKa of fatty acid soap solutions. Langmuir.

[50] Lapitsky Y., Eskuchen W.J., Kaler E.W. (2006). Surfactant and polyelectrolyte gel particles that swell reversibly. Langmuir.

[51] Lapitsky Y., Kaler E.W. (2004). Formation of surfactant and polyelectrolyte gel particles in aqueous solutions. Colloids Surf. A.

[52] Denuziere A., Ferrier D., Domard A. (1996). Chitosan-chondroitin sulfate and chitosan-hyaluronate polyelectrolyte complexes. Physico-chemical aspects. Carbohydr. Polym..

[53] Fajardo A.R., Piai J.F., Rubira A.F., Muniz E.C. (2010). Time- and pH-dependent self-rearrangement of a swollen polymer network based on polyelectrolytes complexes of chitosan/chondroitin sulfate. Carbohydr. Polym..

